# 

**Published:** 1892-05-14

**Authors:** 


### CONTENTS.

Impotent Oxford and its Melancholy Hospital - - - 97

Passing Topics.—Juvenile Smokers - - - - - - 98

The Doctor's Armchair.—The Appeal of the Insane to Science 99

The Story of the Insane from Year to Year - - 100

Medical History from the Earliest Times. By E. T. Withington, M.A., M.B.Oxon. VIII.—The Earliest Greek Medicine—Homer—Æsculapius - - - - - - - 101

The Institutional Workshop—

Asylum Administration.—A New Departure—On the Special Requirements of Ophthalmic Hospitals - - - - - 109

Furniture and Fittings. — Carrying, Commode, and Bath Chairs, 110; A New Form of Hospital Slop Sink, 111; Excreta or Bed-pan Cage - - - - - - - - - - 112

Practical Points - - - - - - - - - - 112

Everybody's Page - - - - - - - - - - 102

Annotations.—Male and Female Brains—Brain Blood in Men and Women—The *Daily Chronicle* and the Scotch Miners - - - 103

Notes and News - - - - - - - - - - 104

Around the Hospitals—Scraps and Gleanings - - - 105

The Practitioner's Mirror and Retrospect—

Medicine.—The Causation and Pathology of Rickets—Post Pharyngeal Abscess - - - - - - - - - 106

Ophthalmology. — Hyperæmia of the Conjunctiva — Simple Conjunctivitis - - - - - - - - - - 107

Practitioner's Bookshelf.—Catechism Series—Public Health —The Pulse - - - - - - - - - - 108

New Drugs, Appliances, and Things Medical.—Jelly Carnis (Caffyn) - - - - - - - - - - 108

The Student of Medicine. — Appointments—Vacancies—Pass Lists.

### EXTRA SUPPLEMENT.—THE NURSING MIRROR.

En Passant.—St. George's Hospital Nurses—The L.O.S. Examination—The Stockton District Nursing Association—The Welsh Branch of the Q V J.I.N.—Cookery—Nurses for India—Short Items—Nurses at Colney Hatch - - - - - xlv

Ventilation, Disinfection, and Diet. By P. Caldwell Smith, M.D. V.—Temperature of Sick Rooms—Ventilation of Hospitals—Ventilation of Sick Rooms - - - - - xlvi

The Metropolitan and National Nursing Association xlvii

For Reading to the Sick.—"The Vineyard" - - - xlvii

Notes from Australia - - - - - - - - - xlviii

Examination Questions - - - - - - - - xlviii

Death in Our Ranks - - - - - - - - - - xlviii

Experiences of a Trained Nurse in Valparaiso During the Revolution - - - - - - - - - - xlix

Notes and Queries - - - - - - - - - - xlix

Nursing Homes. V.—Charing Cross Hospital - - - - l

Appointments - - - - - - - - - - - l

Everybody's Opinion.—Nurses' Holidays—A Holiday in Switzerland - - - - - - - - - - - l

Wants and Workers - - - - - - - - - - l

Four Months in a Hospital Ward (*continued*) - - - li

Darby and Joan - - - - - - - - - - - lii

